# Effects of *FecB* Mutation on Estrus, Ovulation, and Endocrine Characteristics in Small Tail Han Sheep

**DOI:** 10.3389/fvets.2021.709737

**Published:** 2021-11-22

**Authors:** Xiangyu Wang, Xiaofei Guo, Xiaoyun He, Qiuyue Liu, Ran Di, Wenping Hu, Xiaohan Cao, Xiaosheng Zhang, Jinlong Zhang, Mingxing Chu

**Affiliations:** ^1^Key Laboratory of Animal Genetics, Breeding and Reproduction of Ministry of Agriculture and Rural Affairs, Institute of Animal Science, Chinese Academy of Agricultural Sciences, Beijing, China; ^2^Institute of Animal Husbandry and Veterinary Medicine, Tianjin Academy of Agricultural Sciences, Tianjin, China; ^3^Institute of Genetics and Developmental Biology, The Innovation Academy for Seed Design, Chinese Academy of Sciences, Beijing, China

**Keywords:** sheep, *FecB* gene, estrus, ovulation, reproduction hormones, hormone receptors

## Abstract

The Booroola fecundity gene (*FecB*) has a mutation that was found to increase the ovulation rate and litter size in Booroola Merino sheep. This mutation is also associated with the fecundity of small-tail han (STH) sheep, an important maternal breed used to produce hybrid offspring for mutton production in China. Previous research showed that the *FecB* gene affects reproduction in STH sheep, based on litter size records. However, the effects of this gene on estrus, ovulation, and endocrine characteristics in these sheep remain unclear. Here, we analyzed the traits mentioned earlier and compared them among the three *FecB* genotypes of STH ewes using estrus synchronization. Overall, 53 pluriparous ewes were selected from among 890 STH ewes and subjected to *FecB* genotyping for experiments to characterize estrous and ovulation rates. *FecB* heterozygous (+B) ewes presented an earlier onset of estrus (42.9 ± 2.2 h) and a shorter estrous cycle (17.2 ± 0.2 days) (*P* ≤ 0.05). The ovulation rates increased with the increasing copy number of the B allele (*P* ≤ 0.01). Ovulation time showed no significant differences among the three *FecB* genotypes. The serum concentrations of follicle-stimulating hormone (FSH), luteinizing hormone, estrogen (E_2_), and progesterone (P_4_) were measured in 19 of the ewes. Serum concentrations of E_2_ and FSH dramatically varied around the time of behavioral estrus. In *FecB* mutant homozygous (BB) ewes, E_2_ concentration had two peaks, which were higher (*P* ≤ 0.05) than those of ++ genotypes. FSH concentration of BB ewes was higher (*P* ≤ 0.05) than that of the ++ ewes just after estrus. The expression of the estrogen receptor 1 (*ESR1*) gene in the +B genotype was higher than in the other genotypes. Based on the data for the reproductive performance of STH ewes with the three *FecB* genotypes, our study suggests that the development of follicles in ewes with the B allele is dependent on the response to FSH regulated by E_2_ in the early stage. +B ewes, exhibiting moderate ovulation and litter size and a shorter estrous cycle, can be highly recommended in sheep crossbreeding systems for commercial mutton production. Moreover, this study provides useful information to conserve better and use the genetic resources of STH sheep in China.

## Introduction

The *FecB* gene, also called *BMPR1B* (bone morphogenetic protein receptor type 1B), was initially identified in prolific Booroola Merino sheep. These sheep have a mutation (A746G) in the coding region of the *FecB* gene, leading to the substitution of glutamine for arginine in the protein (1–5). The *FecB* gene was shown to have an additive effect on ovulation rate and an effect on litter size that varies from additive to dominant depending on the genotype (6). Moreover, the *FecB* gene might be associated with a higher concentration of follicle-stimulating hormone (FSH) in peripheral blood during the estrous cycle (7–9). It has been reported that progesterone concentrations in Booroola ewes were 25% higher than in control Merino ewes during days 9–11 of the estrous cycle, although the difference did not reach significance (7, 10). *In vitro*, in the absence or presence of FSH, granulosa cells from BB ewes were found to be less responsive to the activity of *GDF5* and *BMP4*, resulting in higher progesterone secretion (3). In addition, it was suggested that granulosa cells carrying this *FecB* mutation might result in reduced proliferation but earlier responsiveness to LH in small follicles (11, 12). The estrous cycle is an external phenotype that is influenced by reproductive hormones (13). Small-tail han (STH) sheep are well known as a high-fecundity breed. The *FecB* mutation in this breed originates from Central Asian sheep, unlike the mutation in Booroola sheep (14). The average litter size of STH primiparous ewes is 2.00, and that of pluriparous ewes is 2.61. Many reports about frequency distributions of the *FecB* gene described that segregations of this gene are found in STH sheep. The +B and BB genotypes are the dominant genotypes in this breed, and the frequency of the B allele is greater than 0.5 (15). The *FecB* gene was shown to significantly affect litter size in STH sheep and other Chinese sheep breeds (16). Because of their excellent reproductive performance, STH sheep are widely used as the female parent in crossbreeding to improve the production efficiency of commercial mutton sheep. In the modern sheep industry, laparoscopic artificial insemination (AI) is being used to maximize reproductive potential. To achieve successful laparoscopic AI, the sheep breed and the signs of behavioral estrus are key factors to be taken into account (17). Based on research on Booroola Merino sheep, it was assumed that the *FecB* gene affects reproductive performance (4, 18). Unlike Booroola Merino sheep, STH sheep are a breed exhibiting year-round estrus. However, there has been little research on the effects of the *FecB* gene on the phenotypes of estrous time, estrous onset, estrous duration, estrous cycle, and subsequent ovulation time in this sheep breed. Therefore, there is a need to evaluate these factors.

Here, estrus and ovulation time traits were measured in three groups of estrus-synchronized STH ewes with different *FecB* genotypes. Combining estrus and ovulation data, we further analyzed the differences in hormonal patterns of the three genotypes in estrous cycles. By analyzing the expression profiles of hormone receptors in granulosa cells of preovulatory follicles in the different genotypes, we also analyzed how hormones coordinate with their receptors to generate signaling cascades that regulate follicle selection and maturation. This research should provide additional insight into the effect of the *FecB* gene on estrus and ovulation events and benefit future sheep reproductive production in China.

## Materials and Methods

### Animals and Genotyping of *FecB* Gene by TaqMan Assay

Jugular vein blood and litter size data of 890 healthy non-pregnant ewes aged approximately 3 years were collected randomly from a herd in southwest Shandong Province, China. These ewes were genotyped for the *FecB* gene by the TaqMan method. Primer sequences of *FecB*-TaqMan and its probe sequences are listed in Table 1. The 6-μl reaction system contained 1 μl of DNA, 3 μl of 2× TaqMan Universal Master Mix II (Thermo Fisher Scientific Inc., Waltham, MA, USA), 0.3 μl of each *FecB*-TaqMan primer, 0.15 μl of two probe primers, and 1.9 μl of H_2_O. Polymerase chain reaction (PCR) amplification was performed on a Roche LightCycler 480 II System (Roche, Basel, Switzerland) under the following PCR conditions: incubation at 95°C for 10 min, followed by 40 cycles of 95°C for 30 s and 60°C for 1 min. The allelic discrimination data were analyzed using endpoint genotyping with the hydrolysis probe protocol.

**Table 1:** TaqMan primer and probe sequences for genotyping and Quantitative PCR primer information.

**Gene**	**GenBank ID**	**Usage**	**Primer sequence (5′–3′)**	
*BMPR1B*	NC_019463.2	Genotyping	Forward primer	CCAGCTGGTTCCGAGAGACA
			Reverse primer	CTTATACTCACCCAAGATGTTTTCATG
			Probe-A	AAATATATCAGACGGTGTTG-MGB
			Probe-G	AAATATATCGGACGGTGTTG-MGB
*FSHR*	NM_001009289.1	Quantitative PCR	Forward primer	CAAAGATCCTCCTGGTCCTGTTC
			Reverse primer	GTTCCTGGTGAAGATGGCGTAG
*LHCGR*	NM_001278566.2		Forward primer	GCTGAGAGTGAACTGAGTGACTG
			Reverse primer	CTGGTTCGGGAGCACATTGG
*INHA*	NM_001308579.1		Forward primer	GACTGGACAGACAGGAGACC
			Reverse primer	AGGACAACGCAGCAGGAG
*ESR1*	XM_027972563.1		Forward primer	TGCCATGTTTCAAACCCATCTTC
			Reverse primer	TCTATGACCAATGACCTCTCTGTG
*CYP19A1*	NM_001123000.1		Forward primer	TCGTCCTGGTCACCCTTCTG
			Reverse primer	CGGTCTCTGGTCTCGTCTGG
*CYP17A1*	NM_001009483.1		Forward primer	CTTACCATTGACAAAGGCACAGAC
			Reverse primer	GCTTAATGATGGCGAGATGAGTTG
*RPL19*	XM_004012836.2		Forward primer	F:AATGCCAATGCCAACTC
			Reverse primer	R:CCCTTTCGCTACCTATACC

Pluriparous ewes (*n* = 53) with similar body conditions were selected and divided into three groups based on the *FecB* genotype (23 wild-type ++ ewes, 16 heterozygote +B ewes, and 14 homozygous mutant BB ewes) for subsequent experiments.

### Experimental Treatment and Design

During the spring, estrus synchronization in these three groups was performed using a controlled internal drug release device (CIDR) (EAZI-BREED® CIDR® Sheep and Goat Device, Pfizer Animal Health, Auckland, New Zealand) with 300 mg of progesterone in all ewes for 12 days without any use of exogenous hormones for superovulation. Only 250,000-IU vitamin A and 25,000-IU vitamin D were injected to protect the vaginal epithelium when the CIDR was inserted. Estrus synchronization was performed on ewes twice with an interval of 14 days. In the first synchronization of estrus in 53 sheep of the three genotypes, estrus response, estrus onset, estrus duration, ovulation time, ovulation rate, and hormonal patterns in these estrous cycles were recorded. In the second experiment, cumulus cells were collected from 12 ewes. All ewes were maintained in a barn with food and water *ad libitum*.

### Estrus Determination

**Figure 1:**
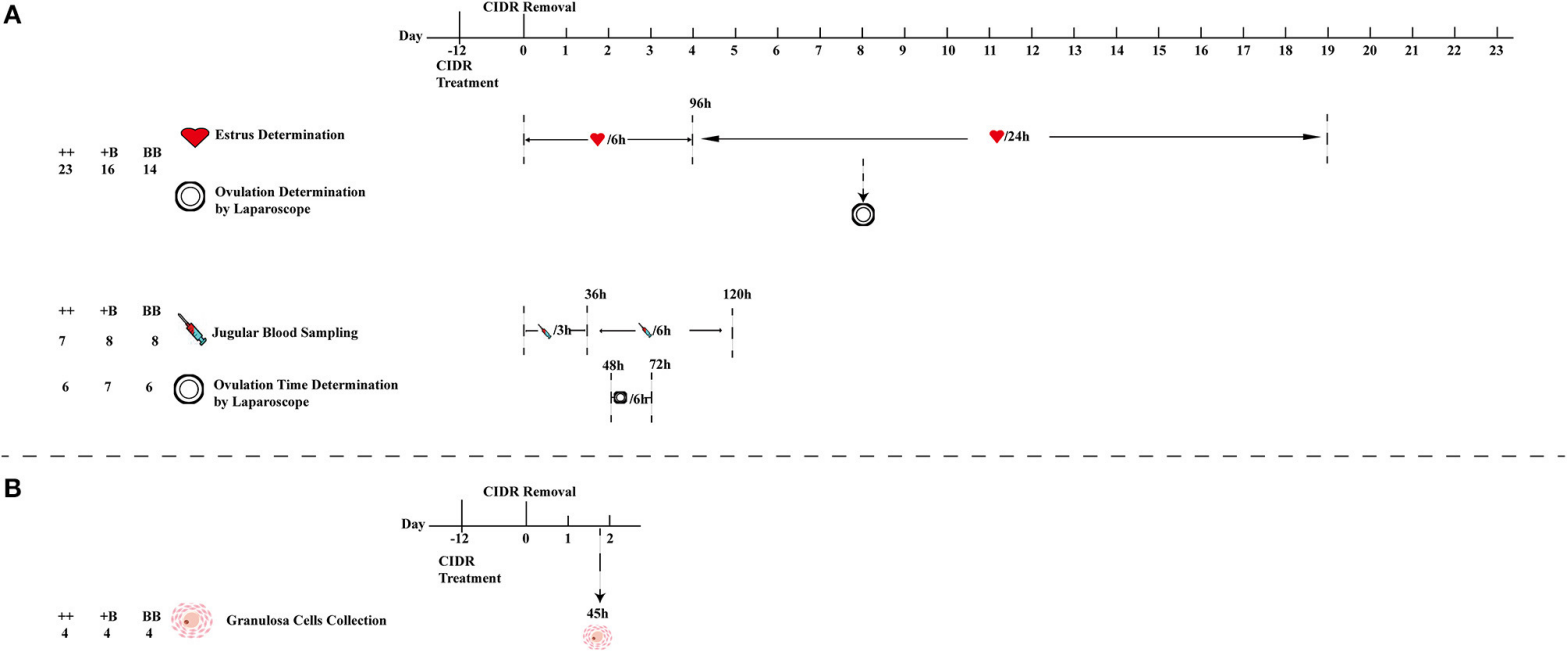
Schematic workflow of estrus and ovulation determination, blood sampling, and granulosa cell collection. **(A)** First estrus synchronization of 53 ewes (23 ++, 16 +B, and 14 BB) for estrus and ovulation rate determination, 23 (7 ++, 8 +B and 8 BB) ewes from 53 ewes were selected for blood sampling, and 19 (6 ++, 7 +B and 6 BB) ewes from 53 ewes for ovulation time and rate determination. **(B)** Second estrus synchronization of 12 ewes for cumulus cell collection from follicles (>3 mm) at 45 h after CIDR removal.

After removal of the CIDR, eight rams with proven high serving capacity were selected as teasers wearing an apron around their hypogastrium to detect estrus in the ewes. The 53 experimental ewes (23 ++, 16 +B, 14 BB) and rams were raised separately. CIDRs were removed at 6:00 a.m. Teasing was initially performed every 6 h (at 6:00, 12:00, 18:00, and 24:00) for 96 h (19–22). Teasing was subsequently performed only once a day at 6:00 a.m. until the initiation of the next estrus. Following established definition and procedures, traits of estrus response, estrus onset, and estrus duration of ewes of the three groups in the first estrous cycle were recorded and calculated (22). The first estrus was defined from the ewes' first accepted mount, and the estrous cycle was defined as the interval from the last refused mount to the next one. Details of the experimental process of estrus determination are presented in Figure 1.

### Ovulation Determination

We randomly selected 6 ++ ewes, 7 +B ewes, and 6 BB ewes from among the 53 ewes of the three experimental groups to determine the ovulation time and rate by a modified version of the method reported by Romano et al. (22, 23). Laparoscopy was performed to determine ovulation at 48, 54, 60, 66, and 72 h after CIDR removal. The intervals from CIDR removal and estrus onset to first ovulation were respectively defined as the times elapsed from CIDR removal and estrus onset to the time halfway between the first appearance of preovulatory follicles and their disappearance. The intervals from CIDR removal and estrus onset to the last ovulation were respectively defined as the times elapsed from CIDR removal and estrus onset to the time halfway between the last appearance of preovulatory follicles and their disappearance. The ovulation rate (the number of corpora lutea on both ovaries) was also recorded for all 53 sheep in the three groups using laparoscopy on day 8 after CIDR removal (Figure 1).

### Blood Sampling and Hormonal Assays

Blood samples for testing hormone levels were collected from 7 ++, 8 +B, and 8 BB ewes from among the 53 ewes at the same time to determine the time of ovulation in the same batch. After removal of the CIDR at 06:00 a.m., 10 ml of jugular vein blood was collected in BD SST tubes (BD, San Diego, CA, USA) every 3 h for a 36-h period (24). From 36 to 120 h, blood was collected every 6 h. Jugular vein blood was kept at room temperature for 30 min, followed by centrifugation at 1,500× *g* for 10 min (25). The concentration of follicle-stimulating hormone (FSH) was determined using a commercial FSH [^125^I]-labeled radioimmunoassay (RIA) kit (B03-FSH; Beijing North Institute of Biological Technology, BNIBT, Beijing, P. R. China). The sensitivity of the kit was <1.0 mIU/ml. The coefficient of variation (CV) in each batch was <10%, and the CV across the batch was <15%. Luteinizing hormone (LH) was quantified using a commercial LH [^125^I]-labeled RIA kit (B04-LH; BNIBT). The kit sensitivity was 1.0 mIU/ml, and intra- and inter-batch CVs were <10 and <15%, respectively. The estradiol (E_2_) concentration was tested using an E_2_ RIA kit (B05-E2; BNIBT), the sensitivity of which was <2 pg/ml. The CVs for each batch and between the batches were <10 and 15%, respectively. Progesterone (P_4_) was quantified using RIA kits (B08-P; BNIBT) with a sensitivity of <0.1 ng/ml, intra-batch CV of <10%, and inter-batch CV of <15%. To ensure the accuracy of hormone detection, triplicate assays were performed for each sample. The details are presented in Figure 1.

### Granulosa Cell Collection and Real-Time Quantitative Polymerase Chain Reaction

Ewes (4 ++, 4 +B, and 4 BB) were used for estrus synchronization. At 45 h after CIDR removal, cumulus–oocyte complexes were aspirated from follicles > 3 mm in diameter with a syringe containing Dulbecco's phosphate-buffered saline (Thermo Fisher Scientific Inc.). Then, the cumulus–oocyte complexes were washed with Dulbecco's phosphate-buffered saline and digested with hyaluronidase. After 5 min of centrifugation at 2,000 rpm, the granulosa cells were gathered and lysed in 1 ml of Trizol (Thermo Fisher Scientific Inc.). The lysate was immediately frozen in liquid nitrogen and stored at −80°C for further RNA extraction.

Total RNA from cumulus cells was isolated following the Trizol protocol. One microgram of RNA was reverse-transcribed with PrimeScript RT Reagent Kit (TaKaRa, Shiga, Japan). Real-time quantitative PCR was performed on the LightCycler 480 Real-Time PCR system (Roche, Switzerland) with the SYBR Premix Ex Taq kit (TaKaRa), as described (26). Primer sequences for quantitative PCR are listed in Table 1. Ribosomal protein L19 (RPL19) was used as an internal reference gene, and the 2^−ΔΔCt^ method was used to determine the relative expression levels in messenger RNA analysis (27).

### Data Analysis

Statistical analysis was performed using the generalized linear model Statistical Analysis System release 8.12 (SAS Institute Inc., NC, USA). All data are presented as the mean ± standard error of the mean, and P ≤ 0.05 was considered significant. *P* ≤ 0.01 was considered extremely significant. The statistical analyses of genotype frequency, allele frequency, and polymorphism information content were performed with reference to the formulas reported by Guo et al. (28). Traits in estrus determination, ovulation time, hormone concentrations in serum, and gene expression were continuous data. The normality of these continuous data was analyzed using the Shapiro–Wilk normality test. One-way analysis of variance was used to analyze the effect of *FecB* mutations on these normally distributed data. The data of the first estrus and estrus cycle were not normally distributed, so the Kruskal–Wallis nonparametric test was applied. Repeated-measures analysis of variance was adopted to analyze how hormonal (FSH, LH, E_2_, and P_4_) concentrations changed in different *FecB* genotypes over time. The estrus response was a binary variable, so Pearson's Chi-squared test was adopted to analyze the estrus response. Litter size and ovulation, traits measured as counts, are usually modeled by the Poisson distribution, so we analyzed the genotypic effects on litter size and ovulation rate through a generalized Poisson regression with log–link function using the generalized linear model GENMOD procedure of SAS. For litter size data, we set *Y*_*j*_ = 0, 1, …, ∞ to be the litter size of individual sheep. The expectation (*E*) and the variance of litter size were calculated as *E*(*Y*_*j*_) = *var*(*Y*_*j*_) = μ_*j*_, where μ_*j*_ = exp(α_0_ + *X*_*j*_β + *Z*_*j*_γ), α_0_ is the intercept, β is a 3 × 1 vector for parity, and γ is a 3 × 1 vector for genotype. The ovulation data from 53 and 19 sheep were analyzed separately, letting *Y*_*j*_ = 0, 1, …, ∞ be the ovulation rate of an individual. The expectation and the variance of ovulation rate were equivalent, *E*(*Y*_*j*_) = *var*(*Y*_*j*_) = μ_*j*_, where μ_*j*_ = exp(α_0_ + *X*_*j*_β), α_0_ is the intercept, and β is a 3 × 1 vector for genotyping.

## Results

### Characteristics of the *FecB* Gene in Small-Tail Han Ewes and Its Association With Litter Size

Using a TaqMan probe-based method, the allele and genotype frequencies of the *FecB* gene in experimental STH ewes were determined, as presented in Table 2. The frequencies of ++, +B, and BB genotypes were 0.16, 0.46, and 0.38, respectively; accordingly, the frequencies of the + and B alleles were 0.4 and 0.6, respectively. Chi-squared fitness testing showed that *FecB* gene mutations were under Hardy–Weinberg equilibrium in the randomly selected ewes (*P* > 0.05). The polymorphism information content of the *FecB* gene exceeded 0.3, reflecting moderate polymorphism, implying that the selection potential of the sites was considerable. For the polymorphic sites of *FecB*, the results of variance analysis as shown in Table 3 indicated that the litter size increased with increasing copy numbers of the B allele. Genotype and parity were shown to have significant effects on litter size. In each parity group, the mean litter size of the ++ genotype ewes was significantly smaller than in the other two genotypes. With increasing parity, the differences in litter size between BB and +B genotypes decreased. There was no significant difference in litter size between the BB ewes and +B ewes in terms of parity above two.

**Table 2:** Genetic characteristics of *FecB* gene in STH ewes.

**Polymorphic site**	**Genotype**	**Number of samples**	**Genotype frequency**	**Allele**	**Allele frequency**	**χ^2^(P)**	**Polymorphism information content**
*FecB*	++	142	0.16	+	0.40	0.61 (0.44)	0.47
	+B	413	0.46	B	0.60		
	BB	335	0.38				

**Table 3:** Litter size of STH ewes in different parities and *FecB* genotypes.

**Genotype**	**Litter size (Mean ± SEM)**			
	**First**	**Second**	**Third**	**Overall mean**
++	1.77 ± 0.08 (115)^A^	1.73 ± 0.09 (73)^A^	2.05 ± 0.22 (20) ^A^	1.78 ± 0.06 (208)^A^
+B	2.04 ± 0.04 (330)^B^	2.25 ± 0.06 (224)^b^	2.76 ± 0.11 (85) ^B^	2.21 ± 0.03 (639)^B^
BB	2.32 ± 0 0.05 (251)^C^	2.51 ± 0.07 (162)^c^	3.00 ± 0.17 (54) ^B^	2.46 ± 0.04 (467)^C^
	*P* (Genotype) <0.01**			
	*P* (Parity) <0.01**			

Numbers of litter size analyzed for each genotype are indicated in brackets. Values with same capital letter for same column have no significant difference (P > 0.05). Values with different lowercases or capital for same line differ significantly (P ≤ 0.05). Double asterisk (**) indicates extreme significance (P ≤ 0.01).

### Estrus Performance After Estrus Synchronization

**Figure 2:**
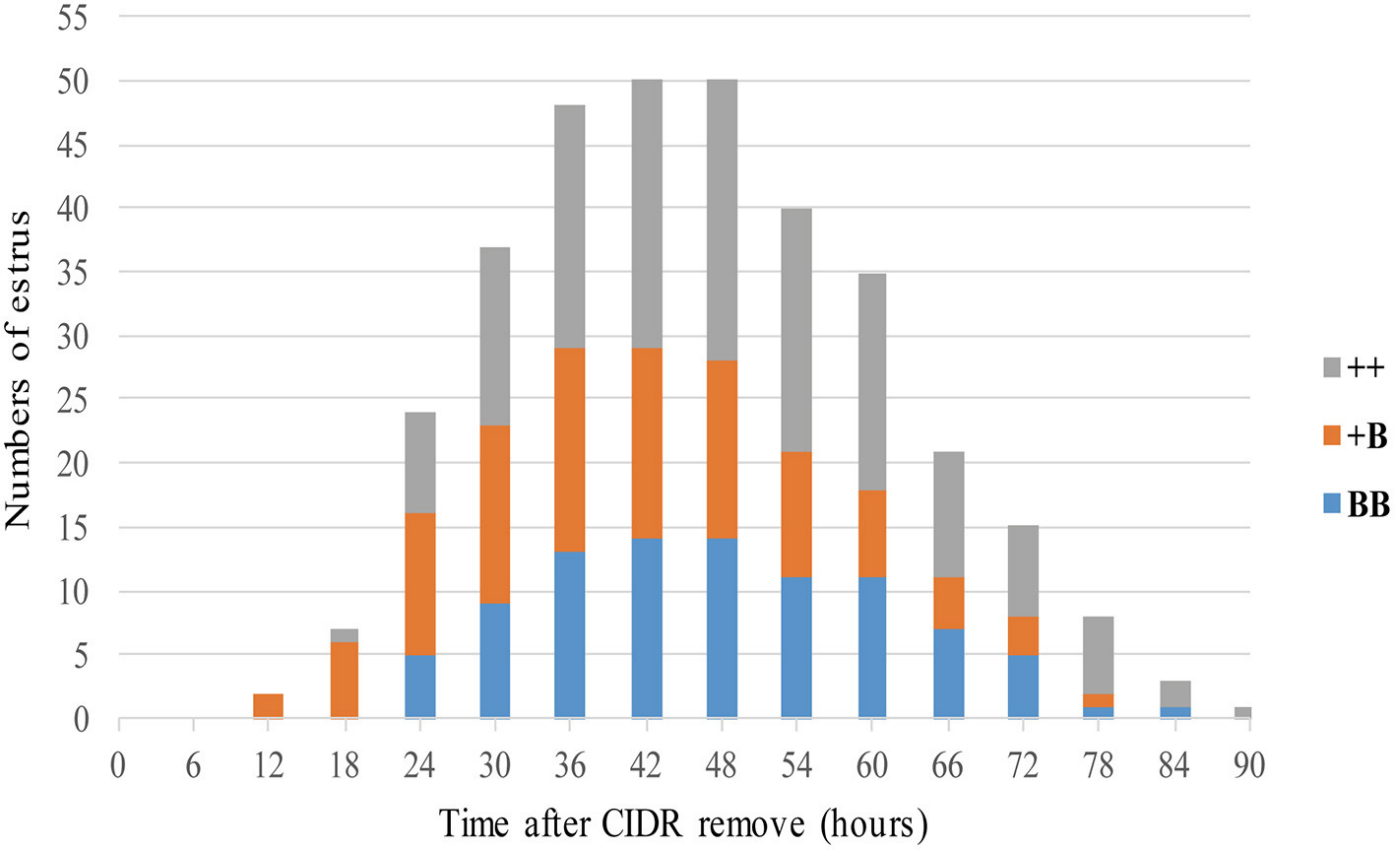
Numbers of ewes in estrus among three *FecB* genotype groups after CIDR removal.

A total of 53 STH ewes of similar ages (2.9 ± 0.1 years) and weights (72.5 ± 1.9 kg) were selected for an estrus determination experiment based on the estrus synchronization technique. After CIDR removal, apart from the estrus duration (32.6 ± 1.4 h), all other estrous cycle indexes were significantly affected by *FecB* (Table 4). The first estrus for +B STH ewes was 23.6 ± 1.9 h from CIDR removal, which was earlier than in the ++ and BB genotypes at 32.6 ± 2.4 and 30.4 ± 1.6 h, respectively (*P* < 0.05). The cumulative estrus response is shown in Figure 2; all ewes presented estrus. The STH ewes with the +B genotype had an earlier response to estrus synchronization treatment, whereas the response of BB STH ewes had a narrower distribution.

**Table 4:** Estrus and ovulation comparison in STH ewes of three *FecB* genotypes.

**Genotype**	**++**	**+B**	**BB**	**Total**
Number of samples	23	16	14	53
Age (y)	2.8 ± 0.1	2.8 ± 0.2	3.0 ± 1.1	2.8 ± 0.1
Weight (kg)	73.8 ± 2.7	73.0 ± 4.6	70.1 ± 3.1	72.5 ± 1.9
Estrus response	23	16	14	53
First estrus (h)	32.6 ± 2.4^a^	23.6 ± 1.9^b^	30.4 ± 1.6^a^	29.3 ± 1.3
Estrus onset (h)	51.5 ± 2.0^a^	42.9 ± 2.2^b^	50.0 ± 1.7^a^	48.5 ± 1.3
Last estrus (h)	65.0 ± 2.4^a^	56.3 ± 2.9^b^	63.4 ± 2.9^ab^	61.9 ± 1.6
Estrus duration (h)	32.4 ± 2.3	32.6 ± 2.3	33.0 ± 3.1	32.6 ± 1.4
Estrus cycle (d)	17.9 ± 0.1^a^	17.2 ± 0.2^b^	17.9 ± 0.2^a^	17.7 ± 0.1
Ovulation Rate	1.09 ± 0.02^A^	2.38 ± 0.13^b^	3.07 ± 0.35^c^	2.02 ± 0.17

*Values with same lowercase for same line have no significant difference (P > 0.05). Values with different lowercases or capital for same line differ significantly (P ≤ 0.05) or extreme significantly (P ≤ 0.01)*.

The onset of estrus in +B STH ewes was 42.9 ± 2.2 h from CIDR removal, which was also earlier than in ++ and BB genotypes with 51.5 ± 2.0 and 50.0 ± 1.7 h (*P* < 0.05). Compared with 17.9 ± 0.1 and 17.9 ± 0.2 days in the STH ewes with ++ and BB genotypes, a shorter estrous cycle of 17.2 ± 0.2 days was observed in the ewes with the +B genotype. The findings suggested that STH ewes with *FecB* heterozygosity (+B) may exhibit an earlier first estrus, earlier estrus onset, and a shorter estrous cycle.

### Ovulation Times and Rates in Small-Tail Han Ewes

Ovulation times and rates in STH ewes among the three genotype groups were observed laparoscopically. The mean interval from CIDR removal to first ovulation in STH ewes was 58.1 ± 2.7 h (Table 5). We noted that the interval from CIDR removal to first ovulation for +B STH ewes appeared to be shorter (52.2 ± 4.4 h), albeit not significantly (*P* = 0.30). The mean intervals from first estrus and estrus onset to first ovulation in STH ewes were 26.3 ± 2.0 and 9.7 ± 1.2 h, respectively. The three *FecB* genotype groups did not differ significantly in any of the variables related to ovulation time. For the ewes with *FecB* mutation, the ovaries did not expel eggs simultaneously. Endoscopy performed every 6 h to estimate the time of ovulation indicated that all ovulations occurred in a period no longer than 6 h. The ovulation rate was measured successfully in the 53 STH ewes (Table 4). The ovulation rate of BB ewes (3.07 ± 0.35) was significantly higher than that of +B ewes (2.38 ± 0.13; *P* ≤ 0.01). Meanwhile, the ovulation rate of +B ewes was also highly significantly higher than that of ++ ewes (1.09 ± 0.02; *P* ≤ 0.01). The *FecB* mutation significantly influenced the ovulation rate of STH ewes.

**Table 5:** Ovulation traits of STH ewes in three *FecB* genotypes.

**Items**	**Genotype**		
	**++**	**+B**	**BB**
Number of samples	6	7	6
Interval from CIDR removal to first ovulation(h)	63.0 ± 5.8	52.2 ± 4.4	58.0 ± 3.3
Interval from estrus onset to first ovulation(h)	9.5 ± 2.9	10.2 ± 0.7	9.3 ± 2.0
Interval from CIDR removal to last ovulation(h)	63.3 ± 5.8	55.8 ± 4.4	62.0 ± 3.6
Interval from estrus onset to last ovulation(h)	9.8 ± 3.1	13.8 ± 3.4	13.3 ± 2.3
Ovulation Rate	1.17 ± 0.41^a^	2.43 ± 0.98^B^	2.00 ± 0.63^b^

*Values with same lowercase for same line have no significant difference (P > 0.05). Values with different lowercases or capital for same line differ significantly (P ≤ 0.05) or extreme significantly (P ≤ 0.01)*.

### Serum Concentrations of P_4_, Follicle-Stimulating Hormone, Luteinizing Hormone, and E_2_ During a Synchronized Estrous Cycle in Small-Tail Han Ewes

**Figure 3:**
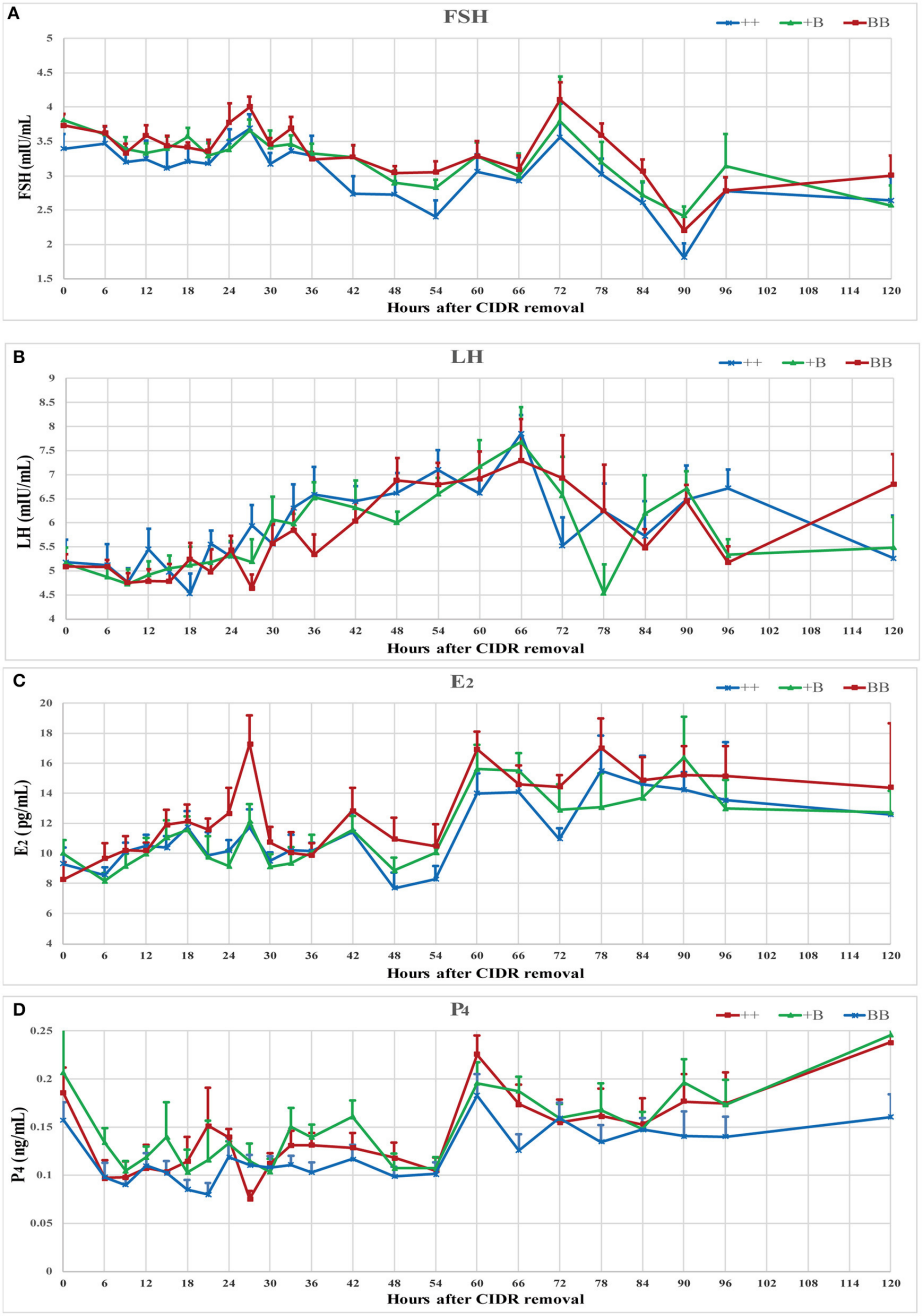
Endocrine profiles of reproductive hormone (FSH, LH, E_2_, and P_4_) in ewes of three *FecB* genotypes during a synchronized estrous cycle. **(A)** Mean ± SEM of serum FSH (mU/ml), **(B)** Mean ± SEM of serum LH (mU/ml), **(C)** Mean ± SEM of serum E_2_ (pg/ml), and **(D)** Mean ± SEM of serum P_4_ (pg/ml).

The serum concentrations of FSH, LH, E_2_, and P_4_ were measured during an intact synchronized estrous cycle in 7 ++, 8 +B, and 8 BB ewes for 5 days after CIDR removal. Data presented in Figure 3 show the mean endocrine profiles of these ewes. Upon CIDR withdrawal, the P_4_ concentration rapidly dropped to a low level. E_2_ and FSH concentrations started to increase and peaked at approximately 27 h, which was aligned with the first estrus time. At 60 h after CIDR removal, the peaks of E_2_ and P_4_ dropped before the LH peak. FSH concentration peaked at 72 h after the LH peak. These four hormone concentrations peaked over 60–72 h.

**Figure 4:**
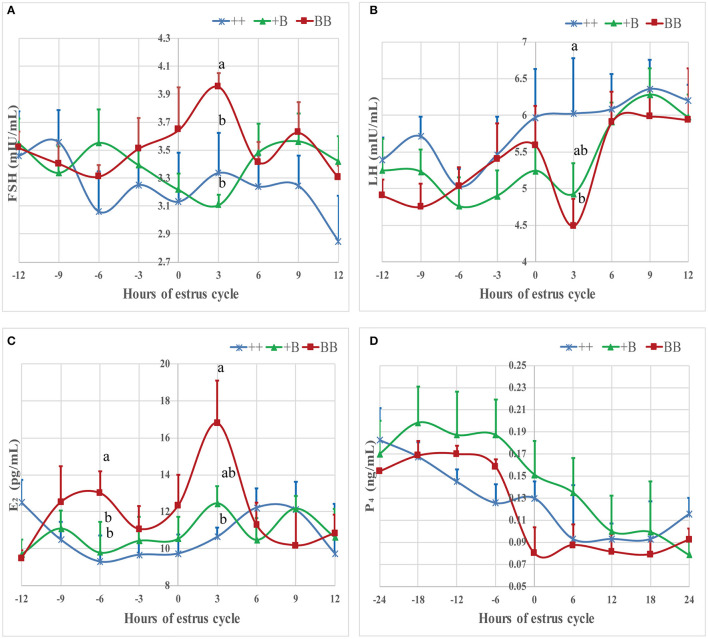
Mean ± SEM of serum FSH, LH, E_2_, and P_4_ concentration before and after initiation of first estrus (hour 0 is initiation of first estrus) in different FecB genotype ewes. Values with same capital letter for same column have no significant difference (*P* > 0.05). Values with different lowercases or capital for same line differ significantly (*P* ≤ 0.05). **(A)** Mean ± SEM of serum FSH (mU/ml), **(B)** Mean ± SEM of serum LH (mU/ml), **(C)** Mean ± SEM of serum E_2_ (pg/ml), and **(D)** Mean ± SEM of serum P_4_ (pg/ml).

The time of the first estrus of +B STH ewes was earlier than those of the other two genotypes. To investigate the changes of endocrine characteristics in serum before and after estrus, mean FSH, LH, E_2_, and P_4_ concentrations were calculated in relation to their levels of each individual's time of the first estrus (hour 0; see Figure 4). In the three genotypes, P_4_ dropped at around hour 0, whereas P_4_ concentrations in BB ewes were higher than those in +B and ++ ewes. E_2_ increased from hour 0 to 3 in all three genotypes. In BB ewes, E_2_ exhibited two peaks (−6 and 3 h), at which the E_2_ concentrations were higher (*P* ≤ 0.05) than in the ++ genotypes. In ++ and +B ewes, the FSH concentrations dropped before hour 0. The FSH concentrations of ++ and BB ewes increased after hour 0 and peaked at hour 3. BB ewes had significantly higher (*P* ≤ 0.05) FSH concentrations than ++ ewes. LH concentrations increased at −3 h and decreased at 3 h, particularly in BB ewes. LH concentrations at 3 h were lower (*P* ≤ 0.05) than in the ++ ewes, whereas those in +B ewes were intermediate.

### Gene Expression in Granulosa Cells

**Figure 5:**
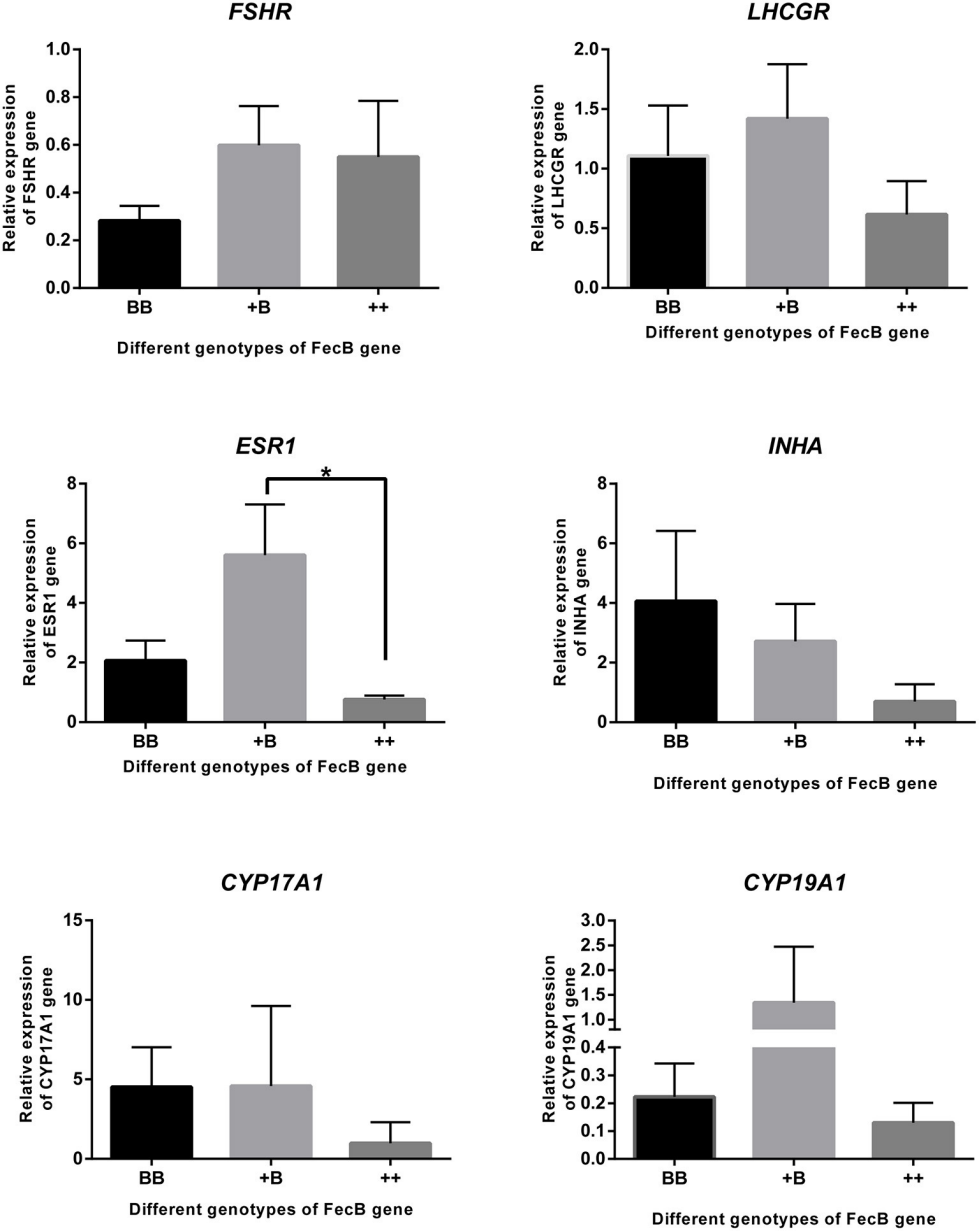
Gene expression in granulosa cells. *FSHR, LHCGR, ESR1, INHA CYP17A1*, and *CYP19A1* genes were quantified using RNA from cumulus cells of follicles (>3 mm) by real-time PCR analysis. Data are mean ± SEM of relative expression level calculated by 2^−ΔΔCt^ method using *RPL19* gene as reference. An asterisk indicates a significant difference (*P* ≤ 0.05) between means from wild-type (++) and heterozygous sheep of *FecB* mutation (+B).

Taking together the results of the temporal trends in reproductive hormone levels and estrus and ovulation times, granulosa cells were derived from preovulatory dominant follicles approximately 45 h after CIDR removal. Total RNA was extracted and subjected to quantitative real-time PCR to analyze the expression of steroidogenic genes, cytochrome P450, family 17, subfamily A, polypeptide 1 (*CYP17A1*) and cytochrome P450, family 19, subfamily A, polypeptide 1 (*CYP19A1*); gonadotropin receptors, follicle-stimulating hormone receptor (*FSHR*) and luteinizing hormone/choriogonadotropin receptor (*LHCGR*); estrogen receptor 1 (*ESR1*); and the inhibin subunit alpha (*INHA*) (Figure 5). Expressions of the genes for the FSH and LH receptors were detected during the preovulatory stage, whereas no significant differences in the expression of these two genes were observed across the three genotypes. The expression of *ESR1* in the +B genotype was higher (*p* ≤ 0.05) than in the wild-type (++) genotype. *ESR1* expression in the BB genotype was higher than that in the wild type, but this did not reach significance. There was a trend for an increase of *INHA* expression with increasing *FecB* mutant copy number (*P* = 0.08). The expression of *CYP17A1* and *CYP19A1*, markers of preovulatory follicles, also appeared at 45 h after CIDR removal when we collected the sample.

## Discussion

STH sheep are derived from ancient northern Mongolian sheep, which are widely raised throughout northern China. The effect of *FecB* mutation in STH matches that identified in Booroola Merino sheep: it can significantly increase the litter size of STH sheep (16, 29, 30). Genetic analysis of the *FecB* gene in this study showed that the three *FecB* genotypes are still not fixed in this STH sheep population collected from a population set aside for conservation. The *FecB* gene showed moderate polymorphisms, meaning that no significant selection response has occurred in the last decade (15, 31). This is good news regarding the protection of genetic diversity and of benefit for efforts to exploit the genetic resources of STH sheep in China.

The *BMPR1B* gene is a member of the transforming growth factor-β superfamily, which is critical for skeletal development, organ formation, and embryo development. Many studies have shown that *FecB* mutations influence ovulation rate, litter size, reproductive endocrinology, adrenal gland size, body mass, follicular development, fetal growth, milk production, and sperm concentration, among others (32–36). With regard to reproductive endocrinology, such mutations can control external estrous characteristics and affect oocyte development. However, little research has been reported on the correlation between *FecB* mutation and estrous characteristics in STH sheep. Here, the estrous indexes of *FecB* heterozygous (+B) STH sheep differed from those in the other genotype groups, presenting an earlier first estrus, earlier estrus onset, and a shorter estrous cycle. However, no differences were observed in ovulation time among the three *FecB* STH genotypes. An investigation of ++ and +B ewes from a three-quarters Romney Marsh × one-quarter Booroola Merino flock after spontaneous estrus induced with progesterone treatment during the reproductive season also found that the *FecB* gene cannot change the duration of ovulation (37). The duration from the first estrus to ovulation was longer in +B STH sheep than in the ++ and BB groups. These results indicate that the *FecB* mutation accelerated the onset of estrus in heterozygote +B STH sheep but had no effect on the onset of ovulation. The +B genotype constituted almost half (0.46) of the total in the STH sheep group; the fecund +B genotype sheep are usually used as maternal parents in crossbreeding programs in commercial mutton production (15). *FecB* gene mutation can result in a change in the estrous phenotype, which is usually the main indicator for determining whether to perform AI (38). When using estrus synchronization with AI in mutton production, we should establish marker-assisted mating and breeding programs. Considering the production cost, prohibition on the use of castrated rams for estrus detection, and the full use of high-breeding-value rams, timed insemination is the optimal mating strategy (39). Combining previous research results and the data of ovulation time from CIDR measured in the three STH *FecB* genotype groups, it was recommended that fixed-time AI be performed between 48 and 60 h after CIDR removal (40, 41).

These results are consistent with previous findings showing that *FecB* mutations appear to have no effect on endocrine profiles (42). In our research, ewes with an *FecB* mutation showed premature estrus. We calculated the individual endocrine data after the time of the first estrus. Before estrus, the ewes with the BB genotype exhibited a peak in E_2_ level, which was the highest among the three genotypes. After estrus, E_2_ exhibited a second peak. During the growing follicle stage, E_2_ downregulates the synthesis and secretion of LH and FSH *via* negative feedback to the hypothalamus and pituitary (43). Our results showed that FSH was not markedly inhibited in the BB genotype; on the contrary, FSH showed significant elevation with a peak at 3 h after estrus. This is in accordance with research on the peripheral FSH concentrations in an F_2_ population from a cross between Booroola Merino and Scottish Blackface sheep during the breeding season (44). As the follicles mature, E_2_ levels peak, and then positive feedback begins, triggering the release of preovulatory LH and an FSH surge (45). The interval from estrus onset to first ovulation suggested that the ewes started estrus approximately 10 h after estrus was detected. LH secretion in ewes peaked at 9 h after estrus. The concentration of FSH was lower than the peak at 3 h after estrus. Increasing FSH of BB ewes accelerated follicle development, meaning that the follicles reached maturity while still having a small diameter. Before ovulation, the FSH concentration decreased during follicle selection, and LH increased to enable the follicles to survive and proceed to ovulation (46). During antral follicle development, the expression of estrogen receptors and *CYP19A1* and E_2_ concentrations significantly increase in granulosa cells (47). The expression pattern of estrogen receptors was similar to that of *CYP19A1* among the three genotypes; in our results, only the expression of *ESR1* was affected by *FecB* mutation. *CYP17A1* and *CYP19A1* play important roles in the steroidogenic pathway. *CYP17A1* expression was higher in the large follicles. LH *via LHCGR* induced the messenger RNA expression of *CYP17A1* in ovaries (48, 49). In the large follicles that we collected, we detected the expression of *CYP17A1* and *LHCGR*. The expression in the ewes with the *FecB* mutation was slightly higher than in the wild type. We also detected the expression of *FSHR* in mature follicles, in agreement with previous findings (50). These two receptors, *FSHR* and *LHCGR*, have been reported to adjust the FSH and LH signaling cascades in granulosa cells and to regulate follicular development and ovulation (51).

Here, STH ewes of ++, +B, and BB genotypes showed significant increases in ovulation rate, and *FecB* mutation carriers had significantly larger litters than the wild-type sheep. These findings are in accordance with previous research reported by Davis et al. (52, 53). Combining the data on litter size, we conclude that the effect of the *FecB* gene on litter size is based on the regulation of the ovulation rate. However, litter size is influenced not only by ovulation rate but also by fertilization, embryonic survival, implantation, placentation, and parity (54). Moreover, *FecB* homozygotes were reported to have a smaller litter size than the heterozygotes in Australian Booroola Merino sheep (55). Therefore, an excessive ovulation rate should be avoided in ewes to prevent lower reproductive efficiency, and sheep with *FecB* heterozygosity exhibiting a higher ovulation rate and larger litter size are recommended for commercial hybrid sheep production in China.

## Conclusion

With the exception of ovulation rate, our results show that the estrus performance of *FecB* heterozygote ewes differed from that of *FecB* homozygote and wild-type ewes, as revealed using estrus synchronization. The development of follicles in ewes with the B allele depended on the response to FSH in the early stage of estrus. *FecB* heterozygote ewes, exhibiting moderate ovulation and litter size and a shorter estrous cycle, can be highly recommended in sheep crossbreeding systems for commercial mutton production. Moreover, the findings obtained in this study should be useful for improving conservation and to better exploit the genetic resources of STH sheep.

## Data Availability Statement

The original contributions presented in the study are included in the article/Supplementary Materials, further inquiries can be directed to the corresponding author/s.

## Ethics Statement

All experimental procedures conducted in this study were performed in accordance with the guidelines set by the Ethics Committee of the Institute of Animal Science, Chinese Academy of Agricultural Sciences, and approved by the committee (no. IASCAAS-AE-03, 12 December 2016).

## Author Contributions

XW, XG, and MC designed this study. XW and XG performed the experimental, processed and analyzed the data, drafted the manuscript, and prepared all the figures and tables. XH and XC collected some serum samples. XW, XG, XZ, and JZ detected the ovulation time and rate. QL, RD, WH, and MC contributed to revisions of the manuscript. All authors read and approved the final manuscript.

## Funding

This work was supported by the National Natural Science Foundation of China (Nos. 31772580, 31501926, and 31902150), Agricultural Science and Technology Innovation Program of China (CAAS-ZDRW202106 and ASTIP-IAS13), Central Public-Interest Scientific Institution Basal Research Fund (No. 2018ywf-yb-2), Earmarked Fund for China Agriculture Research System (CARS-38), Tianjin Agricultural Science and Technology Achievements Transformation and Popularization Program (Grant No. 201704020), Natural Science Foundation of Tianjin (No. 20JCQNJC00630), and the Natural Science Foundation of Jilin Province (20210101376JC).

## Conflict of Interest

The authors declare that the research was conducted in the absence of any commercial or financial relationships that could be construed as a potential conflict of interest.

## Publisher's Note

All claims expressed in this article are solely those of the authors and do not necessarily represent those of their affiliated organizations, or those of the publisher, the editors and the reviewers. Any product that may be evaluated in this article, or claim that may be made by its manufacturer, is not guaranteed or endorsed by the publisher.

## References

[1] Liu Q Pan Z Wang X Wenping HU Ran DI Yao Y. Progress on major genes for high fecundity in ewes. Front Agr Sci Eng. (2014) 1:282–90. 10.15302/J-FASE-201404221951796

[2] Davis GH Montgomery GW Allison AJ Kelly RW Bray AR. Segregation of a major gene influencing fecundity in progeny of Booroola sheep. N Z L J Agr Res. (1982) 67:525–9. 10.1080/00288233.1982.10425216

[3] Mulsant P Lecerf F Fabre S Schibler L Monget P Lanneluc I. Mutation in bone morphogenetic protein receptor-IB is associated with increased ovulation rate in Booroola Mérino ewes. Proc Natl Acad Sci U S A. (2001) 98:5104–9. 10.1073/pnas.091577598 11320249 PMC33171

[4] Wilson T Wu XY Juengel JL Ross IK Lumsden JM Lord EA. Highly prolific Booroola sheep have a mutation in the intracellular kinase domain of bone morphogenetic protein IB receptor (ALK-6) that is expressed in both oocytes and granulosa cells. Biol Reprod. (2001) 64:1225–35. 10.1095/biolreprod64.4.1225 11259271

[5] Souza CJ Macdougall C Macdougall C Campbell BK Mcneilly AS Baird DT. The Booroola (FecB) phenotype is associated with a mutation in the bone morphogenetic receptor type 1 B (BMPR1B) gene. J Endocrinol. (2001) 169:1–6. 10.1677/joe.0.169r001 11312159

[6] Fogarty NM. A review of the effects of the Booroola gene (FecB) on sheep production. Small Rumin Res. (2009) 85:75–84. 10.1016/j.smallrumres.2009.08.003

[7] Xia Y O'Shea T Murison R Mcfarlane JR. Concentrations of progesterone, follistatin, and follicle-stimulating hormone in peripheral plasma across the estrous cycle and pregnancy in merino ewes that are homozygous or noncarriers of the Booroola gene. Biol Reprod. (2003) 69:1079–84. 10.1095/biolreprod.102.005512 12773419

[8] McNatty KP Fisher M Collins F Hudson NL Heath DA Ball K. Differences in the plasma concentrations of FSH and LH in ovariectomized Booroola FF and ++ ewes. J Reprod Fertil. (1989) 85:705–13. 10.1530/jrf.0.0850705 2495362

[9] Fleming JS Tisdall DJ Greenwood PJ Hudson NL Heath DA Mcnatty KP. Expression of the genes for alpha inhibin, beta A inhibin and follistatin in the ovaries of Booroola ewes which were homozygotes or non-carriers of the fecundity gene FecB. J Mol Endocrinol. (1992) 8:265–73. 10.1677/jme.0.0080265 1378743

[10] Bindon BM. Reproductive biology of the Booroola Merino sheep. Aust J Biol Sci. (1984) 37:163–89. 10.1071/BI9840163 6440523

[11] Mcnatty KP Heath DA Hudson NL Lun S Juengel JL Moore LG. Gonadotrophin-responsiveness of granulosa cells from bone morphogenetic protein 15 heterozygous mutant sheep. Reproduction. (2009) 138:545–51. 10.1530/REP-09-0154 19535491

[12] Juengel JL Davis GH McNatty KP. Using sheep lines with mutations in single genes to better understand ovarian function. Reproduction. (2013) 146:R111–23. 10.1530/REP-12-0509 23801782

[13] K.A. Raheem. A review of reproductive events in sheep. J Sustain Agric Environ. (2014) 15:258–75.

[14] Notter DR. Genetic aspects of reproduction in sheep. Reprod Domest Anim. (2008) 43:122–8. 10.1111/j.1439-0531.2008.01151.x 18638113

[15] Hua GH Yang LG. A review of research progress of *FecB* gene in Chinese breeds of sheep. Anim Reprod Sci. (2009) 116:1–9. 10.1016/j.anireprosci.2009.01.001 19201555

[16] Chu M Jia L Zhang Y Jin M Chen H Fang L. Polymorphisms of coding region of BMPR - IB gene and their relationship with litter size in sheep. Mol Biol Rep. (2011) 38:4071–6. 10.1007/s11033-010-0526-z 21110108

[17] Gourley DD Riese RL. Laparoscopic artificial insemination in sheep. Vet Clin North Am Food Anim Pract. (1990) 6:615–33. 10.1016/S0749-0720(15)30836-7 2147121

[18] Souza CJ Campbell BK McNeilly AS Baird DT. Bone morphogenetic proteins and folliculogenesis: lessons from the Booroola mutation. Reprod Suppl. (2003) 61:361–70. 14635948

[19] J.E. Romano. Effect of service on estrus duration in dairy goats. Theriogenology. (1993) 40:77–84. 10.1016/0093-691X(93)90342-3 16727295

[20] J.E. Romano. Effects of different stimuli of service on estrus duration in dairy goats. Theriogenology. (1994) 42:875–9. 10.1016/0093-691X(94)90455-R 16727592

[21] Romano JE Benech A. Effect of service and vaginal-cervical anesthesia on estrus duration in dairy goats. Theriogenology. (1996) 45:691–6. 10.1016/0093-691X(95)00415-5 16727831

[22] Romano JE Alkar A Fuentes-Hernández VO Amstalden M. Continuous presence of male on estrus onset, estrus duration, and ovulation in estrus-synchronized Boer goats. Theriogenology. (2016) 85:1323–7. 10.1016/j.theriogenology.2015.12.018 26838465

[23] Romano JE Fernandez Abella D. Effect of service on duration of oestrus and ovulation in dairy goats. Anim Reprod Sci. (1997) 47:107–12. 10.1016/S0378-4320(96)01633-8 9233510

[24] Drouilhet L Taragnat C Fontaine J Duittoz A Mulsant P Bodin L. Endocrine characterization of the reproductive axis in highly prolific Lacaune sheep homozygous for the FecLL mutation. Biol Reprod. (2010) 82:815–24. 10.1095/biolreprod.109.082065 20075395

[25] Bernini P Bertini I Luchinat C Nincheri P Staderini S Turano P. Standard operating procedures for pre-analytical handling of blood and urine for metabolomic studies and biobanks. J Biomol NMR. (2011) 49:231–43. 10.1007/s10858-011-9489-1 21380509

[26] Guo X Wang X Liang B Di R Liu Q Hu W. Molecular cloning of the B4GALNT2 gene and its single nucleotide polymorphisms association with litter size in small tail han sheep. Animals. (2018) 8:160. 10.3390/ani8100160 30241280 PMC6210199

[27] Livak KJ Schmittgen TD. Analysis of relative gene expression data using real-time quantitative PCR and the 2–ΔΔCT method. Methods. (2001) 25:402–8. 10.1006/meth.2001.1262 11846609

[28] Guo XF Hu WP Lang XZ Li QL Wang XY Di R. Two single nucleotide polymorphisms sites in α1-AT gene and their association with somatic cell score in Chinese Holstein cows. J Biol Res. (2017) 24:8. 10.1186/s40709-017-0065-z 28413783 PMC5390408

[29] Chu MX Liu ZH Jiao CL He YQ Fang L Ye SC. Mutations in BMPR-IB and BMP-15 genes are associated with litter size in Small Tailed Han sheep (Ovis aries). J Anim Sci. (2007) 85:598–603. 10.2527/jas.2006-324 17040942

[30] Qi MY Xu LQ Zhang JN Li MO Lu MH Yao YC. Effect of the Booroola fecundity (FecB) gene on the reproductive performance of ewes under assisted reproduction. Theriogenology. (2020) 142:246–50. 10.1016/j.theriogenology.2019.10.038 31711699

[31] Wang W Liu S Li F Pan X Li C Zhang X. Polymorphisms of the Ovine BMPR-IB, BMP-15 and FSHR and Their Associations with Litter Size in Two Chinese Indigenous Sheep Breeds. Int J Mol Sci. (2015) 16:11385–97. 10.3390/ijms160511385 25993301 PMC4463706

[32] Pardeshi VC Maddox JM Kadoo NY Gupta VS. Genetic modulation of the FecB gene expression. ACIAR Proc Series. (2009) 133:57–65.

[33] Smith P Hudson NL Corrigan KA Shaw L Smith T Phillips DJ. Effects of the Booroola gene (FecB(B)) on bodymass, testis development and hormone concentrations during fetal life. J Reprod Fertil. (1993) 98:41. 9038784 10.1530/jrf.0.1080253

[34] Souza CJ Baird DT. The Booroola (FecB) mutation is associated with smaller adrenal glands in young adult ewes. Reprod Biomed Online. (2004) 8:414–8. 10.1016/S1472-6483(10)60925-X 15149564

[35] Kumar S Mishra AK Kolte AP Arora AL Singh D Singh VK. Effects of the Booroola (FecB) genotypes on growth performance, ewe's productivity efficiency and litter size in Garole x Malpura sheep. Anim Reprod Sci. (2008) 105:319–31. 10.1016/j.anireprosci.2007.03.012 17449205

[36] Gootwine E Zenu A Bor A Yossafi S Rosov A Pollott GE. Genetic and economic analysis of introgression the B allele of the FecB (Booroola) gene into the Awassi and Assaf dairy breeds. Livest Prod Sci. (2001) 71:49–58. 10.1016/S0301-6226(01)00240-8

[37] Souza C Moraes J Chagas LM. Effect of the Booroola gene on time of ovulation and ovulatory dynamics. Anim Reprod Sci. (1994) 37:7–13. 10.1016/0378-4320(94)01324-110967242

[38] Godfrey RW Collins JR Hensley EL. Behavioral and endocrine responses of hair sheep ewes exposed to different mating stimuli around estrus. Theriogenology. (2001) 55:877–84. 10.1016/S0093-691X(01)00450-2 11291911

[39] Olivera-Muzante J Fierro S López V Gil J. Comparison of prostaglandin- and progesterone-based protocols for timed artificial insemination in sheep. Theriogenology. (2011) 75:1232–8. 10.1016/j.theriogenology.2010.11.036 21247622

[40] Fierro S Viñoles C Olivera-Muzante J. Long term prostaglandin based-protocols improve the reproductive performance after timed artificial insemination in sheep. Theriogenology. (2017) 90:109–13. 10.1016/j.theriogenology.2016.11.031 28166955

[41] Ezzat AA Ahmed MN Elabdeen MAAEZ Sabry AM. Estrus synchronization in ossimi sheep by progestins. Alex J Vet Sci. (2016) 51:207–14.27793456

[42] Campbell BK Baird DT Souza CJ Webb R. The FecB (Booroola) gene acts at the ovary: in vivo evidence. Reproduction. (2003) 126:101–11. 10.1530/reprod/126.1.101 12814352

[43] Goodman RL Gibson M Skinner DC Lehman MN. Neuroendocrine control of pulsatile GnRH secretion during the ovarian cycle: evidence from the ewe. Reprod Suppl. (2002) 59:41–56. 12698972

[44] Boulton MI Haley CS Springbett AJ Webb R. The effect of the Booroola (FecB) gene on peripheral FSH concentrations and ovulation rates during oestrus, seasonal anoestrus and on FSH concentrations following ovariectomy in Scottish Blackface ewes. J Reprod Fertil. (1995) 103:199–207. 10.1530/jrf.0.1030199 7616490

[45] Juengel JL. How the quest to improve sheep reproduction provided insight into oocyte control of follicular development. J R Soc N Z. (2018) 48:143–63. 10.1080/03036758.2017.1421238

[46] Baird DT Campbell BK. Follicle selection in sheep with breed differences in ovulation rate. Mol Cell Endocrinol. (1998) 145:89–95. 10.1016/S0303-7207(98)00174-9 9922104

[47] Duan H Ge W Yang S Lv J Ding Z Hu J. Dihydrotestosterone regulates oestrogen secretion, oestrogen receptor expression, and apoptosis in granulosa cells during antral follicle development. J Steroid Biochem Mol Biol. (2021) 207:105819. 10.1016/j.jsbmb.2021.105819 33465420

[48] Kamalludin MH Garcia-Guerra A Wiltbank MC Kirkpatrick BW. Trio, a novel high fecundity allele: I. Transcriptome analysis of granulosa cells from carriers and noncarriers of a major gene for bovine ovulation rate. Biol Reprod. (2018) 98:323–34. 10.1093/biolre/iox133 29088317

[49] Guo YX Duan CH Hao QH Liu YQ Li T Zhang YJ. Effect of short-term nutritional supplementation on hormone concentrations in ovarian follicular fluid and steroid regulating gene mRNA abundances in granulosa cells of ewes. Anim Reprod Sci. (2019) 211:106208. 10.1016/j.anireprosci.2019.106208 31785624

[50] Regan SLP McFarlane JR O'Shea T Andronicos N Arfuso F Dharmarajan A. Flow cytometric analysis of FSHR, BMRR1B, LHR and apoptosis in granulosa cells and ovulation rate in merino sheep. Reproduction. (2015) 150:151–63. 10.1530/REP-14-0581 25948249

[51] Wayne CM Fan H-Y Cheng X Richards JS. Follicle-stimulating hormone induces multiple signaling cascades: evidence that activation of rous sarcoma oncogene, RAS, and the epidermal growth factor receptor are critical for granulosa cell differentiation. Molecular Endocrinology. (2007) 21:1940–57. 10.1210/me.2007-0020 17536007

[52] Davis GH. Major genes affecting ovulation rate in sheep. Genet Sel Evol. (2005) 37:S11–23. 10.1186/1297-9686-37-S1-S11 15601592 PMC3226262

[53] Davis GH. Fecundity genes in sheep. Anim Reprod Sci. (2004) 82:247–53. 10.1016/j.anireprosci.2004.04.001 15271457

[54] Lim W Bae H Bazer FW Song G. Stimulatory effects of fibroblast growth factor 2 on proliferation and migration of uterine luminal epithelial cells during early pregnancy. Biol Reprod. (2017) 96:185–98. 10.1095/biolreprod.116.142331 28395342

[55] Prior J Ghalsasi PM Walkden-Brown SW Chavan KM Kulkarni SR Nimbkar C. Smallholder sheep owners' views on the value and management of Deccani crossbred FecB-carrier ewes with a higher twinning percentage: implications for a future introgression extension program. ACIAR Proc Series. (2009) 199–211.

